# Modulating the tension-time integral of the cardiac twitch prevents dilated cardiomyopathy in murine hearts

**DOI:** 10.1172/jci.insight.142446

**Published:** 2020-10-15

**Authors:** Joseph D. Powers, Kristina B. Kooiker, Allison B. Mason, Abigail E. Teitgen, Galina V. Flint, Jil C. Tardiff, Steven D. Schwartz, Andrew D. McCulloch, Michael Regnier, Jennifer Davis, Farid Moussavi-Harami

**Affiliations:** 1 Department of Bioengineering, College of Engineering and School of Medicine, University of Washington, Seattle, Washington, USA.; 2 Department of Bioengineering, Jacobs School of Engineering, University of California San Diego, La Jolla, California, USA.; 3 Division of Cardiology, School of Medicine, University of Washington, Seattle, Washington, USA.; 4 Department of Chemistry and Biochemistry, College of Science, and; 5 Department of Biomedical Engineering, College of Engineering, University of Arizona, Tucson, Arizona, USA.; 6 Department of Medicine, University of California San Diego, La Jolla, California, USA.; 7 Department of Laboratory Medicine & Pathology, University of Washington, Seattle, Washington, USA.

**Keywords:** Cardiology, Cardiovascular disease, Molecular pathology

## Abstract

Dilated cardiomyopathy (DCM) is often associated with sarcomere protein mutations that confer reduced myofilament tension–generating capacity. We demonstrated that cardiac twitch tension-time integrals can be targeted and tuned to prevent DCM remodeling in hearts with contractile dysfunction. We employed a transgenic murine model of DCM caused by the D230N-tropomyosin (Tm) mutation and designed a sarcomere-based intervention specifically targeting the twitch tension-time integral of D230N-Tm hearts using multiscale computational models of intramolecular and intermolecular interactions in the thin filament and cell-level contractile simulations. Our models predicted that increasing the calcium sensitivity of thin filament activation using the cardiac troponin C (cTnC) variant L48Q can sufficiently augment twitch tension-time integrals of D230N-Tm hearts. Indeed, cardiac muscle isolated from double-transgenic hearts expressing D230N-Tm and L48Q cTnC had increased calcium sensitivity of tension development and increased twitch tension-time integrals compared with preparations from hearts with D230N-Tm alone. Longitudinal echocardiographic measurements revealed that DTG hearts retained normal cardiac morphology and function, whereas D230N-Tm hearts developed progressive DCM. We present a computational and experimental framework for targeting molecular mechanisms governing the twitch tension of cardiomyopathic hearts to counteract putative mechanical drivers of adverse remodeling and open possibilities for tension-based treatments of genetic cardiomyopathies.

## Abstract 2

Tuning the molecular mechanisms that govern the twitch tension of cardiomyopathic hearts counteracts mechanical drivers of adverse remodeling.

## Graphical abstract

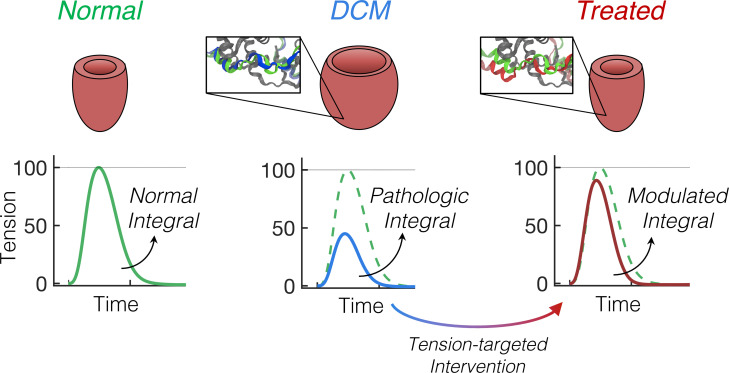

## Introduction

Dilated cardiomyopathy (DCM) is a common and deadly genetic cardiac disorder that affects approximately 1:250 individuals (1) and is typically characterized by enlarged chambers and a thinning of ventricular walls that leads to systolic heart failure (2, 3). DCM is often caused by loss-of-function mutations in genes encoding sarcomere proteins (4–6) that reduce the tension-generating capacity of the myofilaments, which ultimately leads to the adverse ventricular remodeling and heart failure. Currently, treatment options for patients with DCM only delay its progression and do not address the underlying biophysical causes.

**Figure 1:**
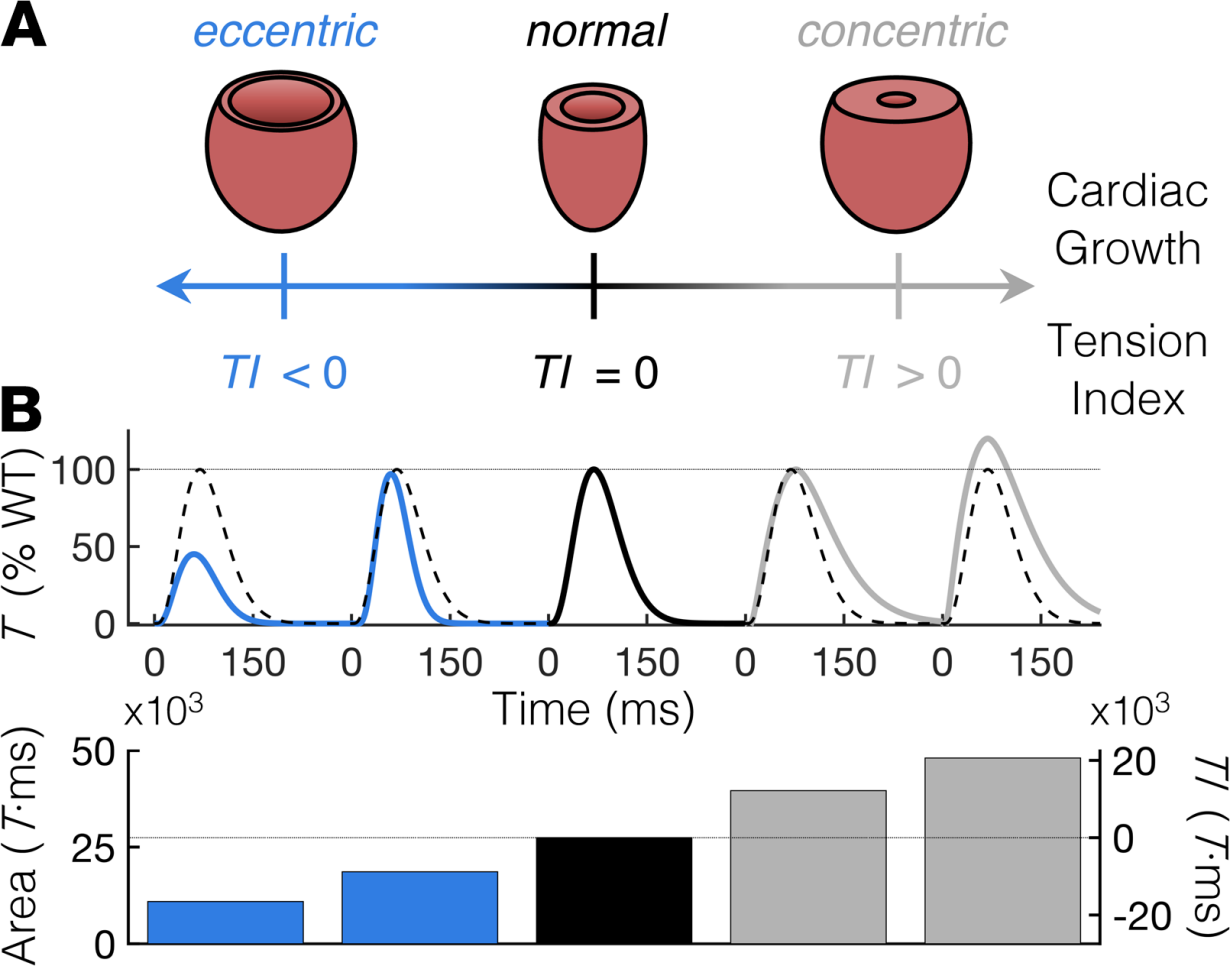
A *TI* based on cardiac twitch *T*-time integrals correlates with the type and severity of myocardial growth. (**A**) Depiction of the relationship between the *TI* and the degree and severity of cardiac growth (modified from ref. 7). The *TI* is determined by subtracting the area under a twitch *T* curve of a cardiomyocyte with perturbed contractility from that of a “normal” or “WT” cardiomyocyte. A positive *TI* correlates with concentric hypertrophy, whereas a negative *TI* correlates with eccentric hypertrophy (7). (**B**) Top panel: Theoretical 300 ms twitch *T* traces of a WT cardiomyocyte (black) and 4 potential variants with altered twitch *T* magnitude and kinetics. All twitches are represented as a percentage of the *T*_peak_ of the WT twitch, which is shown as a dashed black trace with each variant twitch for comparison. Twitches were generated using a simple exponential model, as in ref. 8. Bottom panel: The area under the *T*-time trace (left ordinate) for each corresponding twitch above and the resulting *TI* (right ordinate) calculated as the difference in the area under the twitch *T*-time curve between WT and each variant twitch. Blue and gray correspond to twitches with decreased and increased (respectively) twitch-time integrals compared with WT (black). We note that because the *TI* encompasses contraction and relaxation kinetics, the absolute value of the *TI* does not depend on the peak twitch tension. *T*, tension; *TI*, tension index.

Recently, Davis and colleagues demonstrated that growth and remodeling of cardiomyopathic hearts can be predicted by the duration and magnitude of mechanical tension (*T*) during cardiac twitches (7). The authors systematically perturbed the contractile performance of the sarcomere using a wide variety of genetically engineered murine models and human-induced pluripotent stem cell–derived cardiomyocytes from patients with cardiomyopathies. They found that the twitch *T*-time integral of genetic variants relative to controls, termed the “*T* index” (*TI*), strongly correlates with the type and severity of cardiac growth in each model. More specifically, genetic modifications to the sarcomere that decrease the twitch *T*-time integral (i.e., have a *TI* < 0) strongly correlate with eccentric cardiac growth, whereas modifications that increase the twitch *T*-time integral (i.e., have a *TI* > 0) strongly correlate with concentric cardiac growth (Figure 1A).

The relationship between the *TI* and cardiac growth (Figure 1A) suggested to us that the total net *T* generated during a twitch can be rationally tuned in cardiac muscle with contractile dysfunction to engineer a *T*-based intervention that prevents pathological remodeling. Because the *TI* inherently accounts for altered kinetics of contraction and relaxation relative to normal cardiomyocytes, modulating the *TI* does not inherently require targeting the peak twitch *T* (*T*_peak_; or end-systolic pressure). Figure 1B shows theoretical twitch *T*-time traces (8) to illustrate how variations in twitch amplitude and kinetics compared with “normal” conditions modulate the *TI*. Twitch variants with either increased (gray) or decreased (blue) area under the *T*-time trace compared with a normal (black) twitch therefore have a positive and negative *TI* (respectively), which does not necessarily depend on the *T*_peak_. Thus, the multiple biophysical properties underlying the cardiac twitch that determine the *TI* likely represent multiple tunable targets for modulating the twitch *T* of cardiomyopathic hearts to prevent adverse growth and remodeling.

We (7) and others (9) have found that combining certain gain-of-function protein mutations (i.e., mutations that augment contractility) with the DCM-causing loss-of-function mutations can prevent or reduce the DCM phenotype in murine models. These studies inform on the potential utility of tuning myofilament contractility as a preventative option for genetic cardiomyopathies. However, with a growing number of newly identified cardiomyopathy-causing protein mutations (1), new methodology that can guide the design of treatment options for wide-ranging mutations is needed.

In this work, we combine computational and experimental approaches to target and counteract contractile dysfunction caused by a DCM-associated sarcomere protein mutation. To do so, we employ a transgenic murine model of DCM that is caused by a point mutation in tropomyosin (Tm) at the 230th residue (aspartic acid to asparagine, denoted D230N) (10, 11). This mutation has been found in at least 2 unrelated families with DCM (12). In vitro studies have shown that D230N-Tm decreases the calcium (Ca^2+^) sensitivity of filament sliding and ATPase rates in motility assays (13, 14), decreases the Ca^2+^ affinity of troponin (Tn) C (TnC) (12), and increases the affinity of Tm for actin by nearly 5-fold (14). Moreover, transgenic mice expressing D230N-Tm have significant systolic dysfunction and eccentric hypertrophy by 2 months of age (13). We demonstrate here that the D230N-Tm mutation also significantly decreased the twitch *T* of intact cardiac muscle, thus producing a large, negative *TI* that correlates well with the DCM phenotype found in these mice (13). Using multiscale computational modeling as a guide, we investigate approaches to modulate the *TI* of sarcomeres with D230N-Tm. Our models predict that targeting the Ca^2+^ sensitivity of thin filament activation using the cardiac TnC (cTnC) Ca^2+^-sensitizing variant L48Q (7, 15–20) will augment the twitch *T*–generating capacity of D230N cardiomyocytes. We verify these predictions experimentally by generating a double-transgenic (DTG) mouse model with cardiac expression of both L48Q cTnC and D230N-Tm, and we show that cardiac muscle from DTG hearts had significantly increased contractility compared with D230N hearts. Last, longitudinal echocardiographic monitoring of DTG hearts revealed that the expression of L48Q cTnC in D230N-Tm hearts not only preserved cardiac contractility but also inhibited the development of the DCM phenotype based on functional and morphological echocardiography up to 5 months of age. Thus, our work demonstrates that molecular mechanisms governing cardiac twitch *T*-time integrals can be targeted and tuned to prevent pathological ventricular growth and remodeling in hearts with sarcomeric dysfunction, opening possibilities for other *T*-based therapies.

## Results

### Modulation of the TI of cardiomyocytes with D230N-Tm depended on the inotropic target.

**Figure 2:**
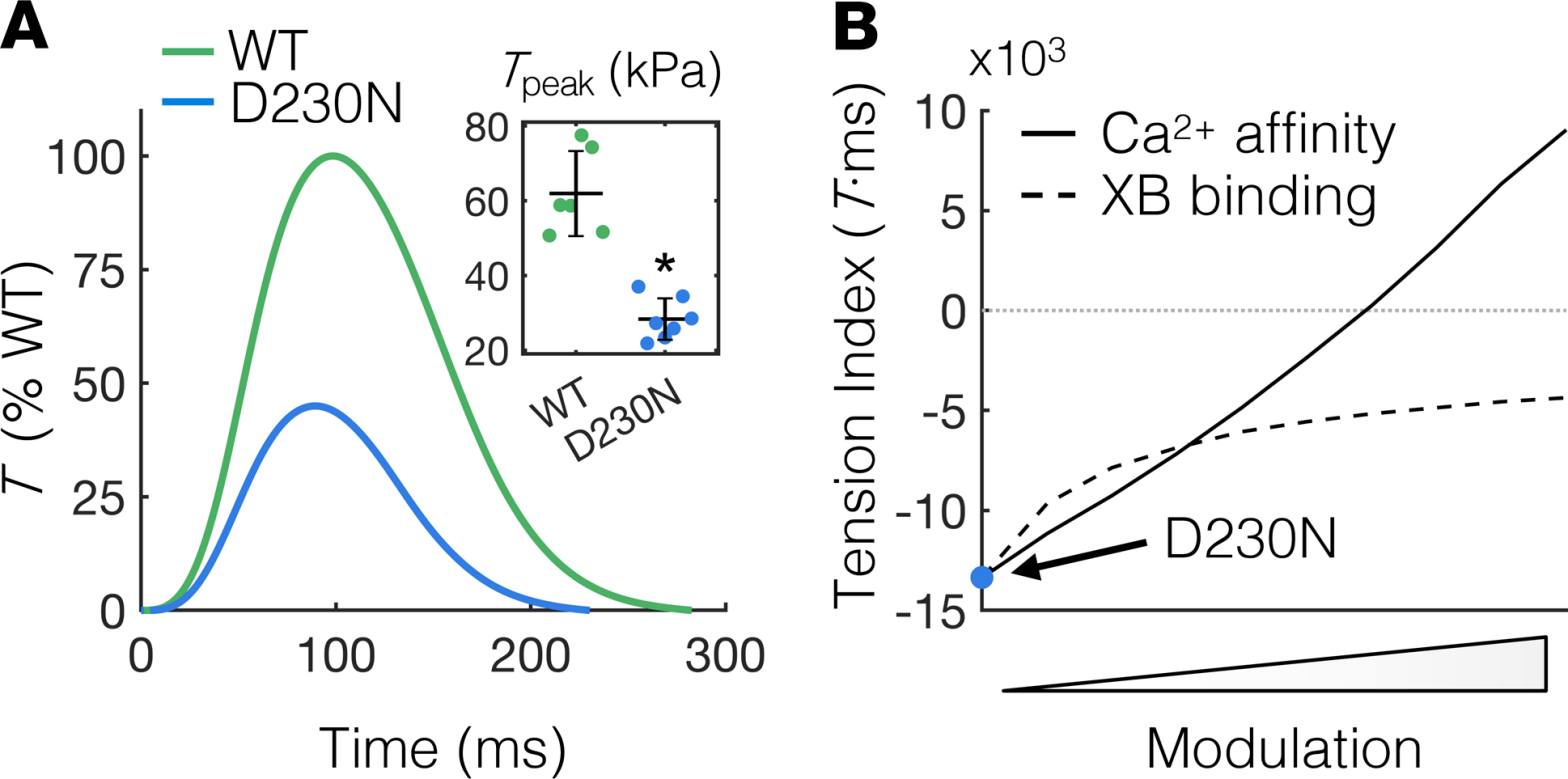
Measuring and modulating the tension index of D230N hearts. (**A**) Average twitch *T* traces of intact trabeculae from WT and D230N hearts as a percentage of WT *T*. The *T*_peak_ of trabeculae from D230N hearts is approximately half that of WT (inset). Sample sizes of *n* = 6 and *n* = 7 for WT and D230N trabeculae, respectively. The error bars of the inset represent SD and *P < 0.01 using a 2-tailed unpaired Student’s *t* test. (**B**) Dependence of the *T* index of simulated D230N twitches on modulation of XB or Ca^2+^ binding. The simulated *T* index for D230N twitches without any modulation is indicated by the blue circle. The rate of XB transition from a weak to a strong (*T*-generating) state was independently increased to simulate D230N twitches with augmented XB binding (dashed line). The affinity of Ca^2+^ for cTnC was also independently increased to simulate twitches of D230N cardiomyocytes with augmented Ca^2+^ sensitivity (solid line). *T*, tension; *T*_peak_, peak twitch tension; XB, cross-bridge; Ca^2+^, calcium; cTnC, cardiac troponin C.

To determine the effects of the D230N-Tm mutation on tissue-level contractility, we measured twitch *T* transients of electrically stimulated intact trabeculae isolated from the right ventricles of WT and D230N-Tm transgenic murine hearts. The twitch *T* of intact trabeculae from D230N hearts was reduced overall compared with WT, with the *T*_peak_ in D230N trabeculae significantly less than that of WT (Figure 2A). Correspondingly, the *TI* of intact trabeculae from D230N hearts, computed as the *T*-time integral relative to WT, was –7.0 × 10^3^ (*T*·ms). The large negative value of the *TI* for D230N trabeculae together with the DCM phenotype observed in these hearts (13) (Supplemental Figure 1; supplemental material available online with this article; https://doi.org/10.1172/jci.insight.142446DS1) further supports the hypothesis from Davis et al. that the *TI* is correlative with ventricular remodeling (7).

In normal cardiomyocytes, any inotropic modulation necessarily affects the *TI*, but the relationship between inotrope and *TI* is likely altered by sarcomere protein mutations that dysregulate contractility. The putatively dysfunctional D230N-Tm (12–14) may supersede thin filament activation altogether, rendering any inotropic modulation ineffective in augmenting the *TI* of D230N hearts. As such, we assessed whether the *TI* of cardiomyocytes containing dysfunctional Tm can be modulated using inotropic intervention and, if so, whether augmented Ca^2+^ sensitivity of thin filament activation or augmented cross-bridge (XB) binding has a greater effect on the *TI*. To do so, we used a computational model of cardiomyocyte contraction (21), as we have done previously (7), to independently and systematically increase either the Ca^2+^ affinity of cTnC or the rate of strong XB attachment in a sarcomere with dysfunctional Tm and calculated the *TI* for each case. Twitches of cardiomyocytes containing dysfunctional Tm were simulated by reducing the rate of transition of Tm from “blocked” to “closed” (21, 22) (see Supplemental Table 1 and Supplemental Figure 2) until *T*_peak_ was reduced by the same amount observed experimentally in intact trabeculae from transgenic hearts containing D230N-Tm (inset of Figure 2A). The resulting *TI* of simulated D230N cardiomyocytes is –13.4 × 10^4^%WT *T*·ms (blue circle in Figure 2B). Progressively increasing the rate of strong XB binding increased the *TI* of simulated D230N cardiomyocytes (Figure 2B, dashed line) until it eventually asymptoted at a value well below 0 (the point at which the *TI* equals that of WT). Conversely, progressively increasing the Ca^2+^ affinity of cTnC increased the *TI* of simulated D230N cardiomyocytes well beyond 0 (Figure 2B, solid line) and did not asymptote for the range of parameters explored here.

### L48Q cTnC induced structural changes in Tn-Tm-actin complexes containing D230N-Tm that likely enhanced thin filament activation.

Our simulated twitches suggest that the *TI* of D230N cardiomyocytes can be greatly increased by augmenting Ca^2+^ binding to cTnC (Figure 2B). When Ca^2+^ binds to cTnC, allosteric interactions in the Tn complex cause the inhibitory peptide region of cTnI to reduce its interaction with actin (23–25), which enables Tm to move from a blocked state to closed and open states that permit varying degrees of XB binding (22). The activated state of the Tn-Tm complex is stabilized by interactions between the switch peptide of cTnI and cTnC (see ref. 24 and references therein). Furthermore, subnanometer changes in intramolecular and intermolecular interactions within a Tn-Tm-actin complex can have great effects on the Ca^2+^ sensitivity of thin filament activation and force generation (16, 26, 27). A cTnC variant engineered to augment this process via increased Ca^2+^ affinity and the cTnC–cTnI interaction is the L48Q cTnC variant (7, 15–20). Although the effects of the L48Q cTnC mutation on the molecular structure of Tn have been previously investigated (16, 19), it is not known if the L48Q cTnC variant retains its Ca^2+^-sensitizing effects on thin filament activation when coupled with the D230N-Tm mutation. Thus, we employed a structural model of the cardiac thin filament (28–31) that includes atomically detailed actin, Tm, and the full Tn complex and incorporated into the model the point mutations D230N in Tm and L48Q in cTnC (Supplemental Figure 3). We then quantitatively assessed structural differences between regulatory units (RUs, defined here as Tn-Tm-actin complexes) containing D230N-Tm with and without L48Q cTnC that may influence the Ca^2+^ sensitivity of thin filament activation.

**Figure 3:**
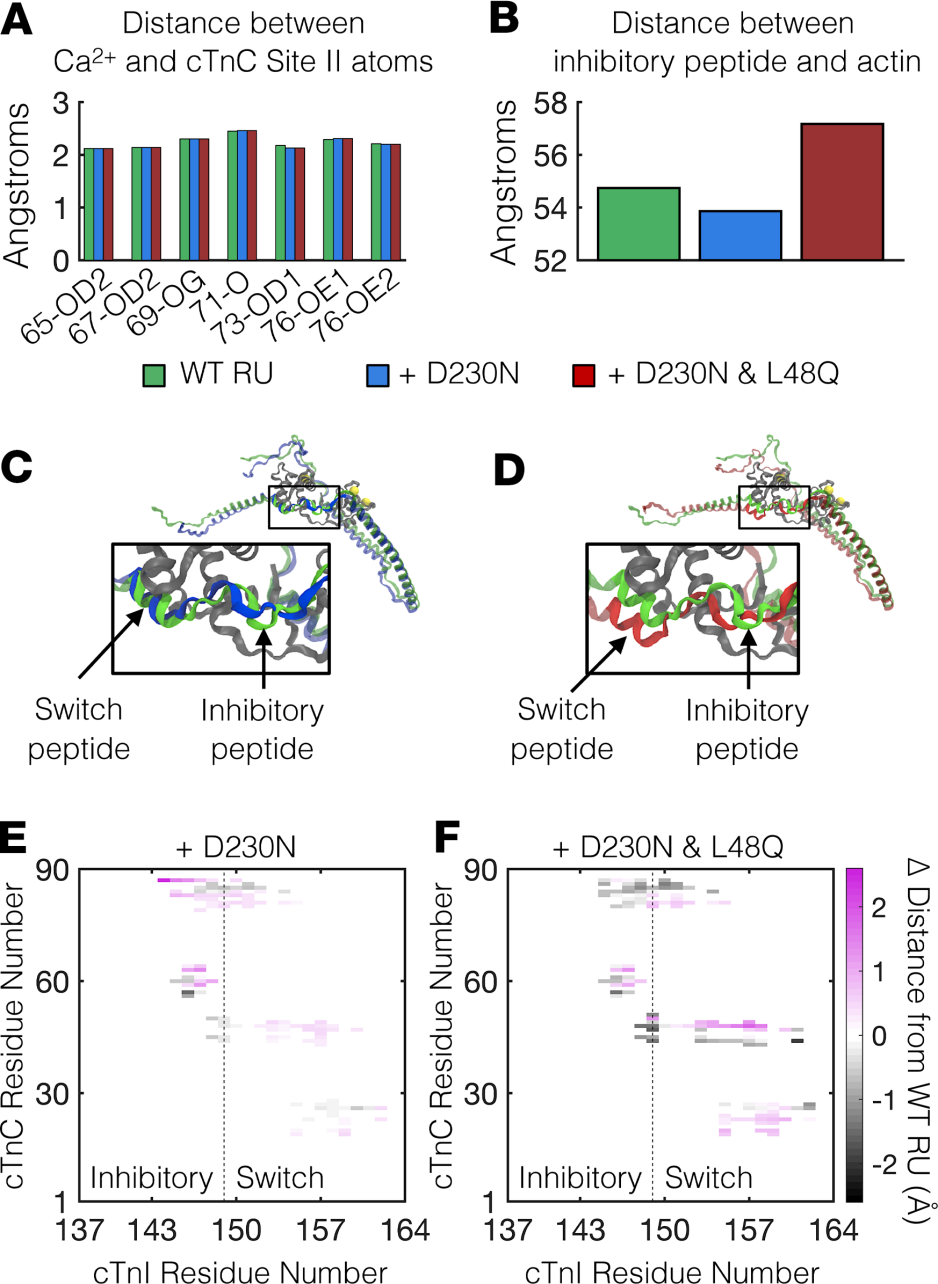
Computational structural analysis of an atomically detailed thin filament with RUs containing D230N-Tm and L48Q cTnC. (**A**) Distances (in Å) between the Ca^2+^ ion and each Ca^2+^-coordinating oxygen atom in site II of cTnC for a WT RU (green), a RU with D230N-Tm (blue) and a RU with both D230N-Tm and L48Q cTnC (red). (**B**) Distances (in Å) between the inhibitory peptide of cTnI and the center of mass of the closest actin monomer for each RU. The color scheme is the same as described for panel **A**. (**C** and **D**) Structural analysis of cTnC and cTnI subunits in the 3 different RUs. The cTnI subunit is shown as green when in the WT RU, blue when in the RU with D230N-Tm, and red when in the RU with both D230N-Tm and L48Q cTnC. cTnC is shown in gray, and Ca^2+^ ions are indicated by the yellow spheres. The switch and inhibitory peptides of cTnI are indicated by the arrows in the close-up insets. (**E** and **F**) Changes in the interactions between cTnC and the inhibitory and switch peptides of cTnI, relative to the WT RU, for the RU containing D230N-Tm (**E**) and the RU containing both D230N-Tm and L48Q cTnC (**F**). The color bar denotes the change in distances (in Å) between cTnC and cTnI residues in each variant RU relative to those in the WT RU. Thus, magenta indicates movement of cTnC–cTnI residues away from one another and black indicates movement of cTnC–cTnI residues toward one another (relative to WT). Residues corresponding to the inhibitory and switch peptides of cTnI are on either side of the vertical dashed line. Tm, tropomyosin; cTnC, cardiac troponin C; RU, regulatory unit; Ca^2+^, calcium; cTnI, cardiac troponin I.

We first investigated the interactions of the ion in the site II Ca^2+^-binding region of cTnC and found that there were no differences in the distances between the Ca^2+^ ion and the Ca^2+^-coordinating atoms in site II of cTnC when comparing the WT RU (Figure 3A, green bars) with RUs containing D230N-Tm with or without L48Q cTnC (Figure 3A, red and blue bars, respectively). However, when Ca^2+^ was bound to site II of cTnC, there were notable differences in the cTn I (cTnI) subunit structure between RU types. Compared with the WT RU, the inhibitory peptide of cTnI was closer to its neighboring actin monomer in the RU containing D230N-Tm, whereas it shifted away from actin in the RU containing both D230N-Tm and L48Q cTnC (Figure 3B). The interactions between the cTnI and cTnC subunits were also different for each RU type. Figure 3, C and D, shows the cTnC (gray) and cTnI subunits for the WT RU (green cTnI) and RUs containing D230N-Tm without L48Q cTnC (blue cTnI in Figure 3C) and with L48Q cTnC (red cTnI in Figure 3D). In the RU with D230N-Tm and WT cTnC, the inhibitory and switch peptides of cTnI generally shifted away from cTnC compared with the WT RU (Figure 3E). Conversely, in the RU containing both D230N-Tm and L48Q cTnC, the inhibitory peptide shifted closer to cTnC, whereas the switch peptide was closer to some cTnC residues and farther away from others compared with the WT RU (Figure 3F). We also note that in an RU containing D230N-Tm, the H1 helix in the I-T arm of cTnI shifted slightly away from cTnC compared with WT, whereas the H1 helix of cTnI in a DTG RU shifted slightly closer to cTnC compared with WT (Supplemental Figure 4). The magnitude of these changes in interaction distances, however, is much smaller (≤0.8 Å) compared with the changes observed in the inhibitory and switch peptides (Figure 3). Together, these results suggest that, when combined with D230N-Tm, L48Q cTnC did not significantly affect the affinity of cTnC for Ca^2+^, but rather strengthened the cTnC–cTnI interaction when Ca^2+^ was bound to site II. Thus, because interactions between the cTnC and cTnI subunits regulate contraction (26, 32–34), our structural model predicts that L48Q cTnC variant likely augments the Ca^2+^ sensitivity of activation of thin filaments containing D230N-Tm by allosterically enhancing the strength of the cTnC–cTnI interaction.

### The reduced contractility of cardiac muscle containing D230N-Tm was prevented by the expression of L48Q cTnC.

**Figure 4:**
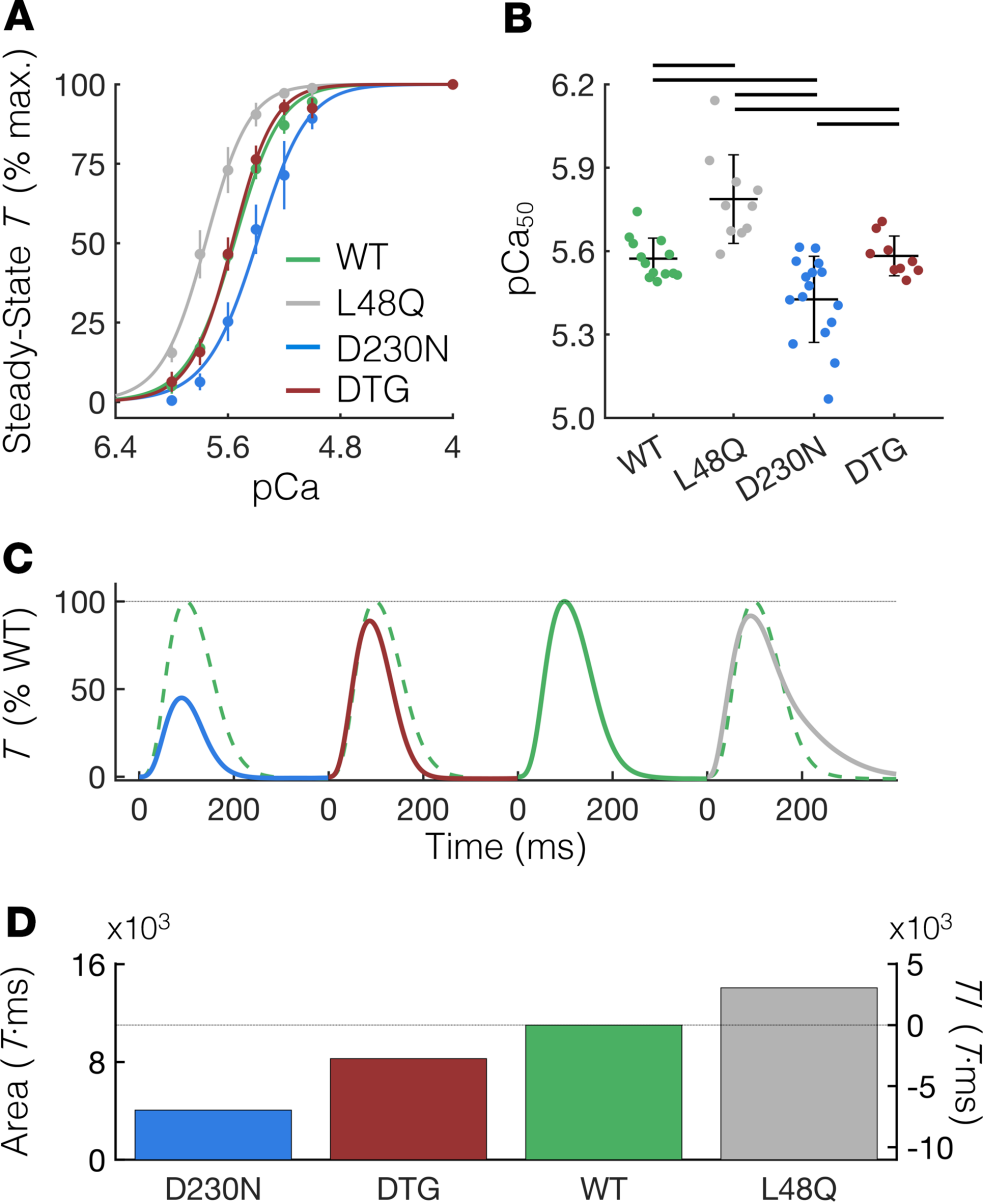
L48Q cTnC prevents contractile abnormalities in cardiac tissue isolated from hearts containing D230N tropomyosin. (**A**) Steady-state *T* as a percentage of the maximum value (at pCa 4.0) of demembranated cardiac muscle measured over a range of extracellular Ca^2+^ concentrations (pCa = –log([Ca^2+^]). The data are fit with the Hill equation (see Methods section) shown by the solid lines. (**B**) The pCa at half-maximal *T* (pCa_50_) of cardiac preparations from D230N hearts is significantly less than all other groups, whereas the pCa_50_ of preparations from L48Q plus D230N DTG hearts is not different from WT. Error bars represent SD. Black lines above the bars indicate *P* < 0.05 between groups using a 1-way ANOVA and a Tukey’s post hoc test of significance. (**C**) Average twitch *T*-time traces (in % WT *T*_peak_) of intact trabeculae for each genotype (same color scheme as panel **A**). The WT *T*-time trace is shown as a dashed green trace against each variant twitch for comparison. (**D**) The area under the *T*-time trace (left ordinate) for each genotype and the resulting *TI* (right ordinate). See Supplemental Table 2 for numerical values. *T*, tension; cTnC, cardiac troponin C; Ca^2+^, calcium; *T*_peak_, peak twitch tension; DTG, double-transgenic.

To demonstrate that the predicted structural effects of L48Q cTnC on RUs containing D230N-Tm (Figure 3) translate into heightened thin filament activation in cardiac muscle preparations, we measured the steady-state *T* (*T*_SS_) generated in membrane-permeabilized cardiac muscle strips (see Methods) isolated from WT, D230N, and D230N plus L48Q DTG mouse hearts for a range of Ca^2+^ concentrations (pCa 9.0 to 4.0). The data were converted to a percentage of the maximum *T*_SS_ value (at pCa 4.0) within each group and fit to the Hill equation. As shown in Figure 4A, the *T*_SS_-pCa curve of cardiac muscle from D230N hearts (blue) was right-shifted compared with WT (green), demonstrating a decrease in Ca^2+^ sensitivity of *T* in D230N hearts. Conversely, the *T*-pCa curve of cardiac muscle from L48Q hearts was left-shifted compared with WT, which is in good agreement with previous studies on the L48Q cTnC variant (17). Most notably, the *T*_SS_-pCa curve of cardiac muscle from DTG hearts was nearly superimposed with that of WT. Correspondingly, the Ca^2+^ concentration at 50% maximum *T* (the pCa_50_) was significantly reduced in cardiac muscle from D230N hearts compared with all other groups (Figure 4B), whereas the pCa_50_ of cardiac muscle from DTG hearts was not different from WT. Neither the Hill coefficient (*n*_H_) nor the maximum *T*_SS_ (at pCa 4.0) had statistically significant differences between any of the groups (Supplemental Table 2).

Next, we assessed whether the improved Ca^2+^ sensitivity of *T*_SS_ of cardiac muscle from DTG hearts compared with D230N hearts translates to increased twitch *T* of intact trabeculae. Figure 4C shows the average twitch *T*-time traces of intact trabeculae isolated from hearts of each genotype, which are shown as a percentage of WT *T*_peak_. The WT *T*-time trace is shown as a dashed green trace against each variant twitch for comparison. We found that the *T*_peak_ of trabeculae from DTG hearts was significantly greater than that of D230N hearts and not different from that of WT (Supplemental Table 2). The kinetics of the twitches were not different between groups, with the exception of significantly decreased relaxation kinetics in trabeculae from L48Q hearts (Supplemental Table 2). We then calculated the area under each twitch *T*-time trace and compared it with WT to determine the *TI* for each variant (Figure 4D). The *TI* of trabeculae from DTG hearts was –2.7 × 10^3^ (%WT *T*·ms), which was greater than approximately 2.5-fold smaller in magnitude than the *TI* of trabeculae from D230N hearts (–7 × 10^3^%WT *T*·ms; see also Supplemental Table 2). These results support the combined predictions of our computational models and demonstrate that the expression of L48Q cTnC in hearts expressing D230N-Tm prevented reductions in both the Ca^2+^ sensitivity of *T* and the twitch *T*–generating capacity caused by the D230N-Tm mutation.

### The DCM phenotype in hearts with D230N-Tm was prevented by expression of L48Q cTnC.

**Figure 5:**
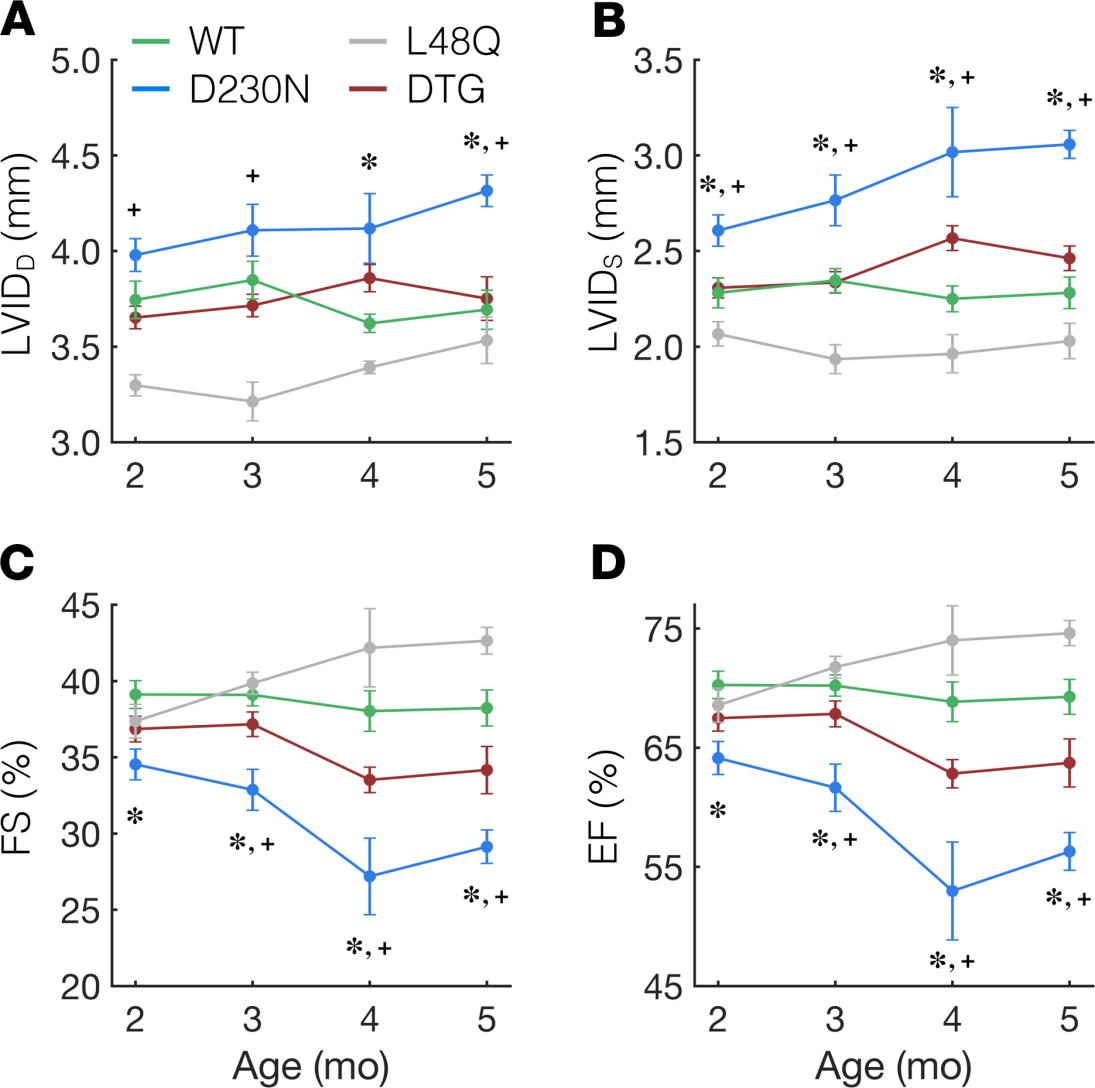
Ventricular remodeling and dysfunction in D230N hearts is prevented by expression of L48Q cTnC. Echocardiographic measurements from mice of 2 to 5 months of age reveal that the left ventricular diastolic (**A**) and systolic (**B**) inner diameter (LVID_D_ and LVID_S_, respectively) of D230N hearts (blue) progressively increase with age, whereas those of DTG hearts (red) do not change with age and are not significantly different from WT (green) at any age. The fractional shortening (**C**) and ejection fraction (**D**) also progressively worsen with age in D230N hearts, whereas those in DTG hearts remain approximately constant and do not significantly differ from WT. * indicates *P* < 0.05 for D230N vs. WT and ^+^ indicates *P* < 0.05 for D230N vs. DTG using a 1-way ANOVA and a Tukey’s post hoc test of significance. Error bars represent the SEM. See Supplemental Table 3 for all values and sample sizes. cTnC, cardiac troponin C; DTG, double-transgenic.

Because the net *T* generated during a twitch of intact trabeculae from DTG hearts was significantly greater than that of hearts containing D230N-Tm (Figure 4), we hypothesized that the degree of ventricular hypertrophy and dysfunction of DTG hearts would be reduced compared with that of D230N hearts. To test this, we used echocardiography to monitor the progression of the DCM phenotype (see Methods) in D230N hearts compared with WT, L48Q, and DTG hearts over a 4-month span (from ages 2 to 5 months). Compared with WT hearts, the left ventricular inner diameters during diastole (LVID_D_, Figure 5A) and systole (LVID_S_, Figure 5B) of D230N hearts progressively increased between 2 and 5 months of age, whereas they were unchanged in DTG hearts. Notably, the LVID_D_ and LVID_S_ of DTG hearts were not significantly different from WT hearts at any time point investigated here (2–5 months). Consistent with this, the LV mass and anterior wall thickness (measured from echocardiogram images, as previously described; refs. 35, 36) of D230N hearts was significantly increased and decreased (respectively) compared with WT by 4–5 months of age, whereas neither the LV mass nor the anterior wall thickness of DTG hearts was different from WT (Supplemental Figure 5). Furthermore, the preserved ventricular dimensions in DTG hearts compared with D230N hearts were accompanied by preserved function. The fractional shortening (FS) and ejection fraction (EF) of D230N hearts progressively worsened from 2–5 months of age, whereas the FS and EF of DTG hearts were approximately constant with age and did not differ significantly from WT hearts at any age (Figure 5, C and D). (See Supplemental Table 3 for numerical values of all echocardiography measurements.) These results demonstrate that hearts containing D230N-Tm progressively developed DCM, whereas the expression of L48Q cTnC in these hearts prevented the development of the DCM phenotype.

## Discussion

The goal of this work was to demonstrate that the total *T* generated during a cardiac twitch is not only an important predictor of myocardial growth and remodeling (7) but also an effective guide for designing approaches to prevent pathological remodeling in hearts with sarcomere dysfunction. To demonstrate this, we used a combined experimental and computational approach to identify tunable molecular interactions in the sarcomere that would enable the modulation of the twitch *T*-time integral of hearts containing the DCM-causing D230N-Tm mutation. Experimentally executing the approach to tune the contractility of D230N-Tm hearts developed through our computational methods, we were able to suppress DCM pathogenesis in murine hearts containing D230N-Tm. Our work demonstrates the ability to prevent pathological growth and remodeling of cardiomyopathic hearts by rationally engineering molecular determinants of the cardiac twitch, opening possibilities for the development of *T*-based treatments of DCM.

A limitation in the *TI* metric as a predictor for cardiac growth is that the absolute value of the *TI* may depend on the experimental or computational conditions in which it is calculated. For example, absolute value of *TI*s calculated from unloaded single-myocyte shortening (as in ref. 7) may vary from *TI*s calculated from isometric tissue-level *T* (this work), which may also vary from *TI*s calculated from ventricular-level pressures. Moreover, *TI*s determined from simulated versus experimentally measured twitches may also attribute to variation in *TI* absolute values. Importantly, however, the *TI* is by definition relative to WT regardless of the experimental or computational conditions in which it is calculated, and we therefore hypothesize that it will be predictive of ventricular and/or cardiomyocyte growth within any given set of conditions. Future work will confirm the scalability and translatability of the *TI* as a global predictor of hypertrophy and remodeling.

Modulation of the *TI* can be achieved through a number of different inotropic interventions, and methods to target the molecular mechanisms of cardiac contractility in the treatment of heart failure have been studied for decades. Small molecules targeting the thick filament that are in human clinical trials (e.g., omecamtiv mecarbil; refs. 37–40), or are being heavily investigated for translation to the clinic (e.g., 2-deoxy-ATP; refs. 41–46), show great promise in treating systolic heart failure. Genetic approaches to engineer the molecular mechanisms of thin filament activation have provided new insights into targeting specific regulators of cardiac contraction for therapeutic applications, including the use of L48Q cTnC as a treatment for systolic heart failure in rat and mouse models of myocardial infarction (15, 17, 18). In DTG mice, the L48Q cTnC variant may not fully correct structural defects in thin filament RUs caused by the D230N mutation in Tm, but instead provides beneficial alterations to intramolecular interactions in Tn that are sufficient to overcome putative D230N-associated dysregulation. Thus, our work and others’ (15, 17, 18) highlight the importance of understanding the molecular interactions in the sarcomere that underlie cardiac twitch properties when developing myofilament-based treatments for contractile dysfunction.

Rationally designed approaches to target contractile dysfunction at the myofilament level require a substantial level of prior knowledge of (a) the consequences of a given mutation on the protein structure and function, (b) the molecular structure-functional effects of the therapeutic under normal circumstances, and (c) potential combinatorial structure-function effects of the mutation and the therapeutic. As the molecular properties of cardiomyopathy-associated protein mutations continue to be characterized, it will therefore be important to catalog mutations based on their effects on cardiac function — particularly how they affect molecular mechanisms that determine twitch *T* and kinetics. Additionally, we note that very few studies of potential treatments for contractile dysfunction to date have investigated effects on twitch kinetics. However, a recent study by Chen et al. revealed that suppressing detyrosination of cardiac microtubules increases the rate of contraction and relaxation without affecting Ca^2+^ transients in failing human hearts (47), which may augment the *TI* of patients with heart failure. Their study, along with the work we present here, highlights the importance of assessing twitch kinetics in potential therapies for heart failure.

Computational models such as those we present here will likely be important tools for predicting and sorting the effects of different sarcomere mutations on twitch *T* and kinetics, as well as facilitating the design of new treatments for genetic cardiomyopathies based on combinatorial effects of the mutation and the treatment. Toward that end, Davis and colleagues found that by classifying mutations that activate or deactivate the thin filament as weak, moderate, or strong, they could construct a model to predict cell behavior for potentially thousands of combinations of known mutations in thin filament proteins (48). Some combinatorial effects between activating and deactivating mutations have already been shown to result in neutralized cardiac function (7, 48), similar to what we present here. Moreover, the ability of computational models to accurately predict structural and functional consequences of pathological protein mutations in the heart enables a completely noninvasive approach to designing therapies. Although we rely heavily on such computational predictions in this work, many of the metrics we use to experimentally confirm those predictions require access to cardiac muscle (e.g., the Ca^2+^ sensitivity measurements and intact trabeculae mechanics). Thus, an important next step in the advancement of computational models is to scale atomistic models to ventricular function such that predictions can be made and corroborated with noninvasive measurements (e.g., echocardiography, MRI) of patients with mutation-causing cardiomyopathies to guide the development of new therapies.

Although significant progress has been made toward using gene therapy to treat heart failure (49–52), current gene delivery approaches can result in low cellular penetrance of the therapy. In the mouse models we present here, the expression of L48Q cTnC was approximately 30% (Supplemental Figure 6). Consequently, when combined with the D230N transgenic mice that express approximately 57% D230N (13) Tm, less than 20% of thin filament RUs included both L48Q cTnC and D230N-Tm. Despite this, we still saw a robust enhancement of cardiac contractility in our DTG mice compared with D230N hearts that is sufficient to prevent the development of the DCM phenotype. Therefore, future work investigating myofilament-targeted treatments for heart failure may not necessarily require high penetrance of the treatment to see a robust enhancement in function (or prevention of dysfunction). However, we note that the (roughly) linear relationship between the *TI* and Ca^2+^ sensitivity modulation predicted by our computational model (solid line of Figure 2B) suggests a dose-dependent response to genetic modification of the myofilaments. Experimental observations support this, as approximately 15% incorporation of L48Q cTnC has been shown to cause little alteration to twitch relaxation kinetics (18), whereas 30% L48Q cTnC incorporation reduced relaxation (Figure 4C and Supplemental Table 2). Furthermore, approximately 45% L48Q cTnC replacement is predicted to significantly increase twitch force and further decrease relaxation kinetics compared with what we report here (7). Thus, the possibility of modulating the *TI* beyond a point of correction and inducing unwanted hypertrophy warrants further investigation. Last, an important next step toward the advancement of therapies targeting cardiac contractility is to elucidate the preventative versus restorative outcomes. Here, we show that inhibiting dysfunction caused by the DCM-associated D230N-Tm mutation by expressing L48Q cTnC prevented the development of DCM, but whether the same approach can be used to *reverse* pathological remodeling remains entirely unknown. Thus, the timing of intervention likely plays a role in determining the outcome of the treatment, and future studies will aid our understanding of when versus how to treat heart failure.

In conclusion, our study presents a framework for employing computational and experimental techniques to rationally tune the molecular mechanisms governing the twitch *T* of cardiomyopathic hearts to counteract mechanical drivers of adverse remodeling, and it has the potential to inform *T*-based therapeutics for genetic cardiomyopathies.

## Methods

### Mice.

Mice were sedated by inhalation of isoflurane, and an i.p. injection of 0.1 mL of heparin was administered to minimize blood clotting in the ventricles. Approximately 4 minutes after the injection of heparin, an i.p. injection of a lethal dose (0.1 mL) of pentobarbital (Beuthanasia-D) was administered.

### Excision of murine hearts.

Hearts were rapidly excised via thoracotomy and immediately immersed in oxygenated (95% O_2_, 5% CO_2_), room temperature Krebs buffer containing (in mM) 118.5 NaCl, 5 KCl, 1.2 MgSO_4_, 2 NaH_2_PO_4_, 25 NaHCO_3_, 1.8 CaCl_2_, and 10 glucose. Hearts were then rinsed via aortic retrograde perfusion with Krebs buffer containing low Ca^2+^ (0.1 mM CaCl_2_) and 20 mM 2,3-butanedione 2-monoxime to minimize contraction and subsequent damage during dissection.

### Intact trabecula mechanics.

Thin, unbranched, and intact trabeculae were carefully dissected from the right ventricular wall and mounted between a force transducer (Cambridge Technology, model 400A) and a length-controlling motor (Aurora Scientific, model 300C). Each end of the trabecula was sutured to custom arms attached to the motor and force transducer made from 22-gauge needles. The trabecula was then submerged in a custom experimental chamber that was continuously perfused with modified Krebs buffer (1.8 mM CaCl_2_) at 30°C. Twitches were elicited by field stimulation with custom platinum plate electrodes at 1 Hz with oscillating polarity. Sarcomere length (SL) was set to 2.0 μm using an inverted stereomicroscope with a ×40 dry objective lens and a ×10 eyepiece. If sarcomeres could not be seen for direct measurement (e.g., if the trabecula was too thick), then an SL of 2.0 μm was assumed to be at trabecula slack length (the length of the trabecula at the onset of passive *T* development). Trabeculae were allowed to pace at 1 Hz for approximately 20 minutes at SL 2.0 μm (and 30°C) and then stretched to SL 2.3 μm for data acquisition.

Continuous twitch *T*-time traces were recorded using custom LabView software at a sampling rate of 1 kHz and were analyzed using custom code with MATLAB software (version 2018a, The MathWorks).

### Permeabilized trabecula mechanics.

Hearts were permeabilized in a “skinning” solution containing 100 mM KCl, 10 mM MOPS, 5 mM EGTA, 9 mM MgCl_2_, either 1 mM dATP or 4 mM ATP (adjusted to pH = 7 with KOH), 1% (by volume) Triton X-100, 1% protease inhibitor (MilliporeSigma P8340), and glycerol at 4°C overnight. Permeabilized trabeculae were then dissected from the right and left ventricles and mounted between a force transducer and motor using custom aluminum T-clips. SL was measured using a Fourier transform of a digitized image of the sarcomeres using an IonOptix camera connected to a ×40 dry objective lens. SL was set to 2.3 μm for the experiments. Trabeculae were submerged in physiological solution at 15°C containing a range of pCa (= –log[Ca^2+^]) from 9.0 to 4.0 and allowed to reach *T*_SS_ at each pCa. *T*_SS_-pCa curves for each genotype were collected and analyzed using custom code with LabView software. Then they were fit to the Hill equation, as *T*_SS_ = *T*_SS,Max_ × [1 + 10*^n^*_H_^×^^(pCa^_50_^–^^pCa)^]^–1^, where *T*_SS,Max_ is the maximum *T*_SS_ (at pCa 4.0), pCa_50_ is the pCa at half-maximal *T*, and *n*_H_ is the Hill coefficient (the slope of the *T*_SS_-pCa relation and a measure of the cooperativity of *T*).

### Echocardiography.

Male and female mice of each genotype from 2 to 5 months of age were used for echocardiographic measurements, as previously described (44). Briefly, animals were lightly anesthetized and held under anesthesia via inhalation of 1% isoflurane in 95% oxygen. Transthoracic echocardiography was performed using Vevo 2100 high-frequency, high-resolution imaging system (VisualSonics) equipped with MS400 MicroScan Transducer. The parasternal short axis view at the mid-papillary level was used to obtain M-mode images for measurements of left ventricular inner diameters at the end of diastole and end of systole, FS, and EF. The FS was calculated from these data using the relationship 100 × (LVID_d_ – LVID_s_)/LVID_d_, where LVID_d_ and LVID_s_ are the left ventricular inner diameters at the end of diastole and systole, respectively.

### Calculation of the TIs.

Similar to Davis et al. (7), the *TI* was calculated based on the *T*-time integral from either intact trabecula mechanics or the computationally simulated cardiac twitches. Twitch *T*-time traces were normalized to the maximum WT value for each genotype. The time integral of the twitch *T* was then calculated using a point-by-point integration method based on cumulative trapezoidal approximations using MATLAB software (version 2018a, The MathWorks). The *TI* was then calculated as the difference between the time integral of either parameter for each genotype relative to WT. (The *TI* for WT is therefore 0 by definition.)

### Computational simulations of cardiac twitches.

Similar to what we have done previously (7), we simulated twitches of cardiomyocytes from hearts with DCM-causing mutations in the sarcomere by modifying the parameters in the Negroni-Lascano model (21) to fit our experimental measurements of cardiac twitches. As shown in Supplemental Figure 2, the model consists of 6 actomyosin states: no Ca^2+^ bound to Tn (TS), Ca^2+^ bound to Tn with no XBs (TSCa_3_), Ca^2+^ bound to Tn with weak XB attachment (TSCa_3_~), Ca^2+^ bound to Tn with strong (*T*-generating) XB attachment (TSCa_3_*), no Ca^2+^ bound to Tn with strong (*T*-generating) XB attachment (TS*), and no Ca^2+^ bound with weak XB attachment (TS~).

Original parameter values and initial conditions from the Negroni-Lascano model (4) were used to simulate “WT” twitches. To simulate D230N-Tm twitches, we reduced the transition rate from TSCa_3_ to TSCa_3_~ (parameter f, see Supplemental Table 1) by 43.5% such that the *T*_peak_ was decreased by the same amount compared with WT (~50%) as observed experimentally. Y_b_, Z_b_, Y_r_, and Z_r_, which represent Ca^2+^ association and dissociation from Tn, were varied to assess the effects of Ca^2+^ modulation on sarcomeres with the simulated D230N-Tm. Z_p_, and Y_p_, the transition rates between TSCa_3_~ and TSCa_3_*, and g, the transition rate from TSCa_3_~ to TSCa_3_, were varied to assess the effects of cross-bridge modulation on cardiomyocytes with simulated D230N-Tm. Forward rates (Y_b_, Z_r_, and Y_p_) were progressively increased by factors of 2–10 (with the exception of Y_b_, which was increased by a maximum of 9-fold to allow for model convergence), and reverse rates (Z_b_, Y_r_, Z_p_, and g) were simultaneously decreased by factors of 0.9–0.5 to assess a range of Ca^2+^ and XB modulation (Supplemental Table 1). SL was set to 1.8 μm. Model equations were implemented using MATLAB software (version 2018b, The MathWorks) and solved using a forward Euler method for 1000 ms under isometric conditions.

### Molecular simulations of cardiac thin filament structure.

The molecular model of the cardiac thin filament was constructed using the previously described initial low-temperature structure (28–31) that includes actin, Tm, and the full Tn complex (Supplemental Figure 3). Point mutations were integrated into each respective protein using the CHARMM42 program (53) with CHARMM36 parameters, the latest version of the CHARMM force field (54). This substitution is performed by initially deleting the side chain atoms of the respective residue and constructing in the new atoms using the parameters.

The complete structures, including the point mutations, were then explicitly solvated in a water box that allowed at least a 15 Å barrier from all edges of the protein with TIP3P waters using the SOLVATE plugin in VMD1.9.3 (55). The system was ionized to a concentration of 0.15 mol/L using potassium and chloride ions with the AUTOIONIZE plugin in VMD1.9.3. All simulations were performed with NAMD2.12 (56) using CHARMM36 parameters with the SHAKE algorithm. The Particle-Mesh Ewald method was used with a cutoff of 12 Å to calculate all nonbonded interactions between atoms. Each individual system underwent 5000 steps of minimization, slow heating to a temperature of 300 K and at rate of 1 K/ps, and then equilibration in an isobaric-isothermal ensemble at 1 atm and 300 K for 690 ps. With the equilibrated structure, 3 separate 10 ns production runs were performed with randomly generated velocities from a Boltzmann distribution. The average coordinates of each atom within each respective structure were calculated and these average structures were used to calculate all statistics.

The distance between the inhibitory peptide of cTnI and actin was calculated by averaging the distance between the alpha carbons of cTnI residues 137–147 and the center of mass of the closest actin monomer. The cTnI–cTnC residue contact maps were created by extracting distances between each cTnC–cTnI residue using VMD1.9.3 for each structure. Residues that were less than or equal to 10 Å apart in the WT RU were used to analyze the change in those distances upon introducing the D230N-Tm mutation and the L48Q cTnC mutation. The distances between each of the cTnC–cTnI residue pairs from the WT structure were subtracted from that of each mutated structure (D230N and D230N plus L48Q) and then plotted using MATLAB (version 2018a, The MathWorks).

### Statistics.

All experimental data were collected using custom data acquisition software developed using LabView and were analyzed using MATLAB (version 2018a, The MathWorks) software and built-in statistical packages. Unless stated otherwise, error bars represent SEM. A 1-way ANOVA with a Tukey’s post hoc test of significance was used to compare values across multiple genotype groups, unless stated otherwise.

### Study approval.

All animal experiments were done in accordance with protocols approved by the University of Washington Institutional Animal Care and Use Committee and followed the *Guide for the Care and Use of Laboratory Animals* (National Academies Press, 2011). Adult male and female mice between 4 and 6 months of age were euthanized following the procedures approved by the Institutional Animal Care and Use Committee for the University of Washington.

## Author contributions

JDP, MR, JD, and FMH designed the study. JDP, KBK, ABM, AET, and GVF performed the experiments. JDP, KBK, ABM, AET, GVF, JD, and FMH analyzed the data. JDP, KBK, ABM, AET, GVF, JCT, SDS, ADM, MR, JD, and FMH helped interpret the results. JCT provided the D230N transgenic murine model. MR, JD, and FMH developed the DTG murine model. JDP wrote the manuscript.

## Supplementary Material

supplemental data

## Version 1 09/15/2020

In-Press Preview

## Version 2 10/15/2020

Electronic publication

## References

[1] McNally EM, Mestroni L (2017). Dilated cardiomyopathy: genetic determinants and mechanisms. Circ Res.

[2] Hershberger RE, Morales A, Siegfried JD (2010). Clinical and genetic issues in dilated cardiomyopathy: a review for genetics professionals. Genet Med.

[3] Hershberger RE, Siegfried JD (2011). Update 2011: clinical and genetic issues in familial dilated cardiomyopathy. J Am Coll Cardiol.

[4] Herman DS (2012). Truncations of titin causing dilated cardiomyopathy. N Engl J Med.

[5] McNally EM, Golbus JR, Puckelwartz MJ (2013). Genetic mutations and mechanisms in dilated cardiomyopathy. J Clin Invest.

[6] Chang AN, Potter JD (2005). Sarcomeric protein mutations in dilated cardiomyopathy. Heart Fail Rev.

[7] Davis J (2016). A tension-based model distinguishes hypertrophic versus dilated cardiomyopathy. Cell.

[8] Tu MS, Daniel TL (2004). Cardiac-like behavior of an insect flight muscle. J Exp Biol.

[9] Li J (2018). Sarcomere-based genetic enhancement of systolic cardiac function in a murine model of dilated cardiomyopathy. Int J Cardiol.

[10] Tardiff JC (2011). Thin filament mutations: developing an integrative approach to a complex disorder. Circ Res.

[11] Tardiff JC (2010). Tropomyosin and dilated cardiomyopathy: revenge of the actinomyosin “gatekeeper”. J Am Coll Cardiol.

[12] Lakdawala NK (2010). Familial dilated cardiomyopathy caused by an alpha-tropomyosin mutation: the distinctive natural history of sarcomeric dilated cardiomyopathy. J Am Coll Cardiol.

[13] Lynn ML, Tal Grinspan L, Holeman TA, Jimenez J, Strom J, Tardiff JC (2017). The structural basis of alpha-tropomyosin linked (Asp230Asn) familial dilated cardiomyopathy. J Mol Cell Cardiol.

[14] Gupte TM (2015). Mechanistic heterogeneity in contractile properties of α-tropomyosin (TPM1) mutants associated with inherited cardiomyopathies. J Biol Chem.

[15] Tikunova SB, Davis JP (2004). Designing calcium-sensitizing mutations in the regulatory domain of cardiac troponin C. J Biol Chem.

[16] Wang D (2012). Structural and functional consequences of the cardiac troponin C L48Q Ca(2+)-sensitizing mutation. Biochemistry.

[17] Feest ER (2014). Thin filament incorporation of an engineered cardiac troponin C variant (L48Q) enhances contractility in intact cardiomyocytes from healthy and infarcted hearts. J Mol Cell Cardiol.

[18] Shettigar V (2016). Rationally engineered Troponin C modulates in vivo cardiac function and performance in health and disease. Nat Commun.

[19] Kekenes-Huskey PM, Lindert S, McCammon JA (2012). Molecular basis of calcium-sensitizing and desensitizing mutations of the human cardiac troponin C regulatory domain: a multi-scale simulation study. PLoS Comput Biol.

[20] Parvatiyar MS, Pinto JR, Liang J, Potter JD (2010). Predicting cardiomyopathic phenotypes by altering Ca2+ affinity of cardiac troponin C. J Biol Chem.

[21] Negroni JA, Lascano EC (2008). Simulation of steady state and transient cardiac muscle response experiments with a Huxley-based contraction model. J Mol Cell Cardiol.

[22] McKillop DF, Geeves MA (1993). Regulation of the interaction between actin and myosin subfragment 1: evidence for three states of the thin filament. Biophys J.

[23] Gordon AM, Homsher E, Regnier M (2000). Regulation of contraction in striated muscle. Physiol Rev.

[24] Cheng Y, Regnier M (2016). Cardiac troponin structure-function and the influence of hypertrophic cardiomyopathy associated mutations on modulation of contractility. Arch Biochem Biophys.

[25] Marston S, Zamora JE (2020). Troponin structure and function: a view of recent progress. J Muscle Res Cell Motil.

[26] Lindert S, Cheng Y, Kekenes-Huskey P, Regnier M, McCammon JA (2015). Effects of HCM cTnI mutation R145G on troponin structure and modulation by PKA phosphorylation elucidated by molecular dynamics simulations. Biophys J.

[27] Wang D (2013). Structural and functional consequences of cardiac troponin C L57Q and I61Q Ca(2+)-desensitizing variants. Arch Biochem Biophys.

[28] Williams MR, Lehman SJ, Tardiff JC, Schwartz SD (2016). Atomic resolution probe for allostery in the regulatory thin filament. Proc Natl Acad Sci USA.

[29] Williams MR, Tardiff JC, Schwartz SD (2018). Mechanism of cardiac tropomyosin transitions on filamentous actin as revealed by all-atom steered molecular dynamics simulations. J Phys Chem Lett.

[30] McConnell M (2017). Clinically divergent mutation effects on the structure and function of the human cardiac tropomyosin overlap. Biochemistry.

[31] Abdullah S (2019). FRET-based analysis of the cardiac troponin T linker region reveals the structural basis of the hypertrophic cardiomyopathy-causing Δ160E mutation. J Biol Chem.

[32] Abbott MB, Dvoretsky A, Gaponenko V, Rosevear PR (2000). Cardiac troponin I inhibitory peptide: location of interaction sites on troponin C. FEBS Lett.

[33] Cheng Y (2014). Computational studies of the effect of the S23D/S24D troponin I mutation on cardiac troponin structural dynamics. Biophys J.

[34] Dewan S, McCabe KJ, Regnier M, McCulloch AD, Lindert S (2016). Molecular effects of cTnC DCM mutations on calcium sensitivity and myofilament activation — an integrated multiscale modeling study. J Phys Chem B.

[35] Gardin JM, Siri FM, Kitsis RN, Edwards JG, Leinwand LA (1995). Echocardiographic assessment of left ventricular mass and systolic function in mice. Circ Res.

[36] Kiatchoosakun S, Restivo J, Kirkpatrick D, Hoit BD (2002). Assessment of left ventricular mass in mice: comparison between two-dimensional and m-mode echocardiography. Echocardiography.

[37] Utter MS, Ryba DM, Li BH, Wolska BM, Solaro RJ (2015). Omecamtiv mecarbil, a cardiac myosin activator, increases Ca2+ sensitivity in myofilaments with a dilated cardiomyopathy mutant tropomyosin E54K. J Cardiovasc Pharmacol.

[38] Greenberg BH (2015). Safety and tolerability of omecamtiv mecarbil during exercise in patients with ischemic cardiomyopathy and angina. JACC Heart Fail.

[39] Shen YT (2010). Improvement of cardiac function by a cardiac myosin activator in conscious dogs with systolic heart failure. Circ Heart Fail.

[40] Winkelmann DA, Forgacs E, Miller MT, Stock AM (2015). Structural basis for drug-induced allosteric changes to human β-cardiac myosin motor activity. Nat Commun.

[41] Regnier M, Rivera AJ, Chen Y, Chase PB (2000). 2-deoxy-ATP enhances contractility of rat cardiac muscle. Circ Res.

[42] Kadota S (2015). Ribonucleotide reductase-mediated increase in dATP improves cardiac performance via myosin activation in a large animal model of heart failure. Eur J Heart Fail.

[43] Lundy SD (2014). Cell-based delivery of dATP via gap junctions enhances cardiac contractility. J Mol Cell Cardiol.

[44] Nowakowski SG (2013). Transgenic overexpression of ribonucleotide reductase improves cardiac performance. Proc Natl Acad Sci USA.

[45] Moussavi-Harami F (2015). 2-Deoxy adenosine triphosphate improves contraction in human end-stage heart failure. J Mol Cell Cardiol.

[46] Powers JD (2019). Cardiac myosin activation with 2-deoxy-ATP via increased electrostatic interactions with actin. Proc Natl Acad Sci USA.

[47] Chen CY (2020). Depletion of vasohibin 1 speeds contraction and relaxation in failing human cardiomyocytes. Circ Res.

[48] Davis J, Metzger JM (2010). Combinatorial effects of double cardiomyopathy mutant alleles in rodent myocytes: a predictive cellular model of myofilament dysregulation in disease. PLoS One.

[49] Kolwicz SC (2016). AAV6-mediated cardiac-specific overexpression of ribonucleotide reductase enhances myocardial contractility. Mol Ther.

[50] Tilemann L, Ishikawa K, Weber T, Hajjar RJ (2012). Gene therapy for heart failure. Circ Res.

[51] Pleger ST (2011). Cardiac AAV9-S100A1 gene therapy rescues post-ischemic heart failure in a preclinical large animal model. Sci Transl Med.

[52] Hoshijima M (2002). Chronic suppression of heart-failure progression by a pseudophosphorylated mutant of phospholamban via in vivo cardiac rAAV gene delivery. Nat Med.

[53] Brooks BR (2009). CHARMM: the biomolecular simulation program. J Comput Chem.

[54] Vanommeslaeghe K (2010). CHARMM general force field: A force field for drug-like molecules compatible with the CHARMM all-atom additive biological force fields. J Comput Chem.

[55] Humphrey W, Dalke A, Schulten K (1996). VMD: visual molecular dynamics. J Mol Graph.

[56] Phillips JC (2005). Scalable molecular dynamics with NAMD. J Comput Chem.

