# Supplementary material for: Modulating the tension-time integral of the cardiac twitch prevents dilated cardiomyopathy in murine hearts
Source: JCI Insight. 2020 Oct 15;5(20):e142446. doi: 10.1172/jci.insight.142446 (PMC7605524; doi:10.1172/jci.insight.142446)
Supplement: supplemental data [file jciinsight-5-142446-s134.pdf]

Supplemental Material

Powers *et al. "Modulating the tension-time integral of the cardiac twitch prevents dilated cardiomyopathy in murine hearts"*

Supplemental Figures

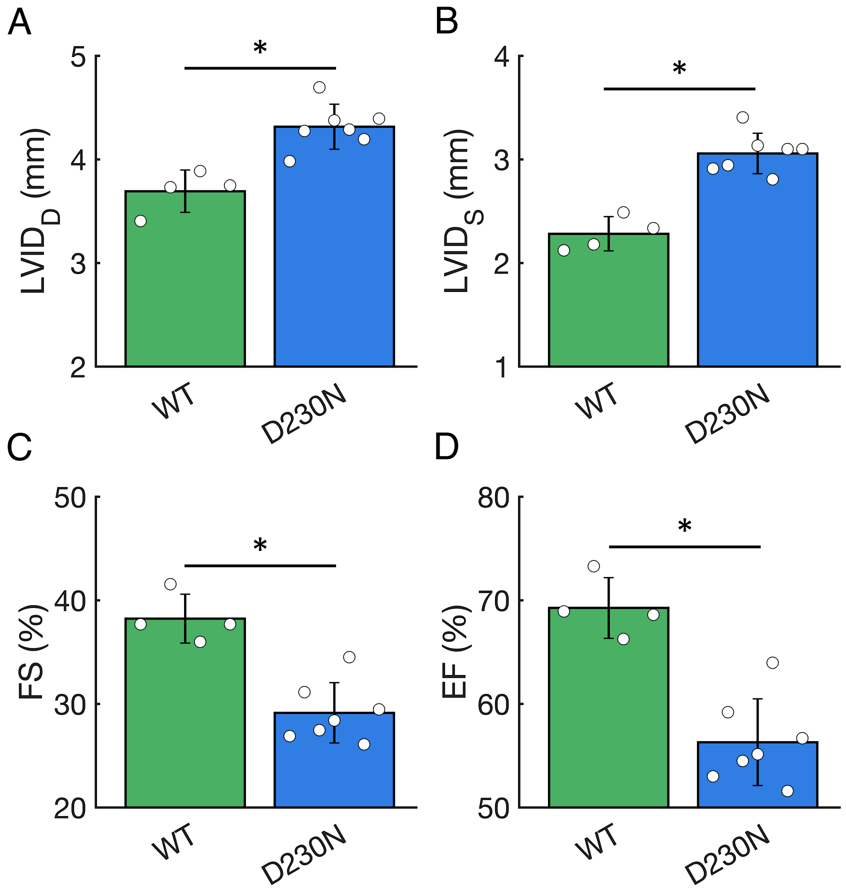

Figure S1. Echocardiographic measurements reveal a DCM phenotype in adult murine hearts with D230N Tm. (**A**) The left-ventricular inner diameter during diastole (LVID$_D$) and (**B**) during systole (LVID$_S$) are significantly greater in hearts with D230N Tm compared to WT. (C) The fractional shortening (FS) and (**D**) the ejection fraction (EF) are significantly reduced in hearts with D230N Tm compared to WT. Error bars represent SD from n = 4 hearts for WT and n = 7 hearts for D230N. Male and female mice were 5 months of age. *$p < 0.005$ using an unpaired student's t-test.

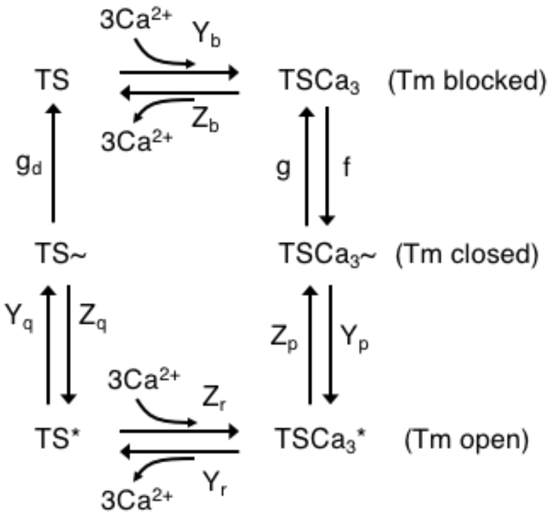

Figure S2. The tension index of twitches with dysfunctional tropomyosin depends on the inotropic target. Schematic of the Negroni-Lascano model of cardiac tension development during twitch transients (schematic modified from Reference 18 in the main text). All parameters are defined in the Methods section of the main text. For clarity, we have labeled the states in which tropomyosin (Tm) is occupied.

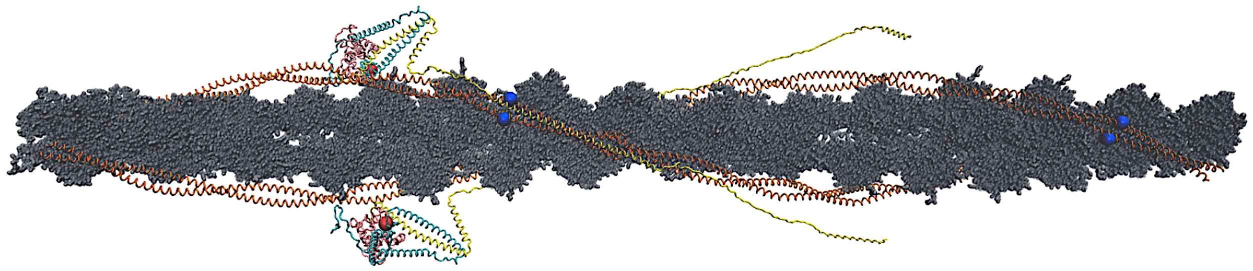

Figure S3. Computational structural model of an atomically detailed cardiac thin filament. The simulated cardiac thin filament consists of F-actin (gray) tropomyosin (orange), troponin C (pink), I (cyan), and T (yellow). The residues of Tm and cTnC where the mutations were incorporated are indicated by the spheres (D230N Tm in blue and L48Q cTnC in red).

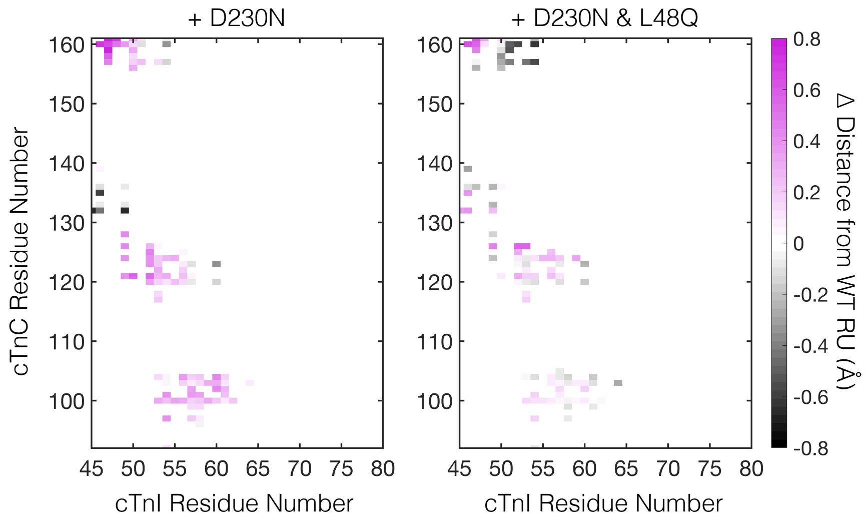

Figure S4. Analysis of the interactions between the H1 Helix in the I-T arm of cTnI and cTnC. Changes in the interactions between the H1 helix of the I-T arm in cTnI and cTnC for a regulatory unit containing D230N Tm (left) and a regulatory unit containing both D230N Tm and L48Q cTnC (right). The colorbar denotes changes in the distances (in Å) between cTnC and cTnI residues in each variant regulatory unit relative to the WT RU.

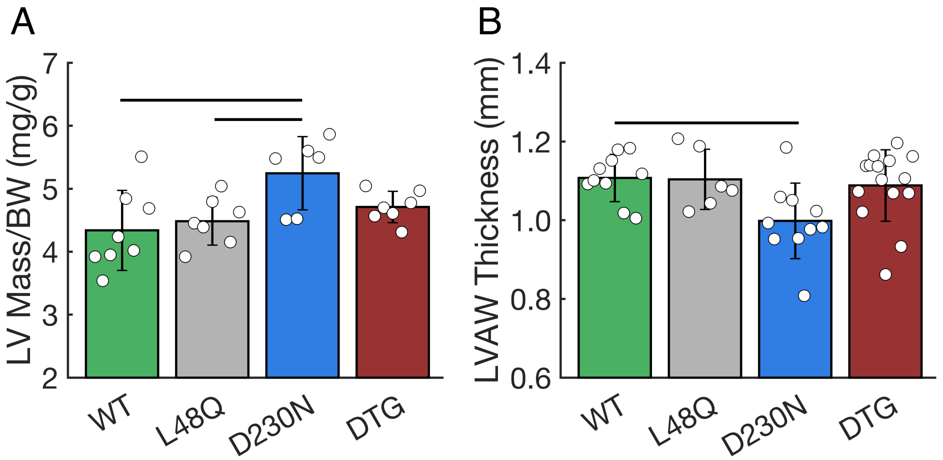

Figure S5. Left ventricular mass and thickness measurements. (**A**) The left ventricular mass (taken from the M mode echocardiogram images and normalized to mouse body weight) is significantly greater in D230N hearts compared to WT and L48Q cTnC controls, while the that of DTG hearts is not different from any group. (**B**) The diastolic left ventricular anterior wall thickness is significantly less in D230N hearts compared to WT, while that of DTG hearts is not different from WT. Error bars represent standard deviation and the black lines above the bars indicate $p < 0.05$ using a one-way ANOVA with a Tukey post-hoc test of significance. Data are pooled from 4–5-month-old male and female mice.

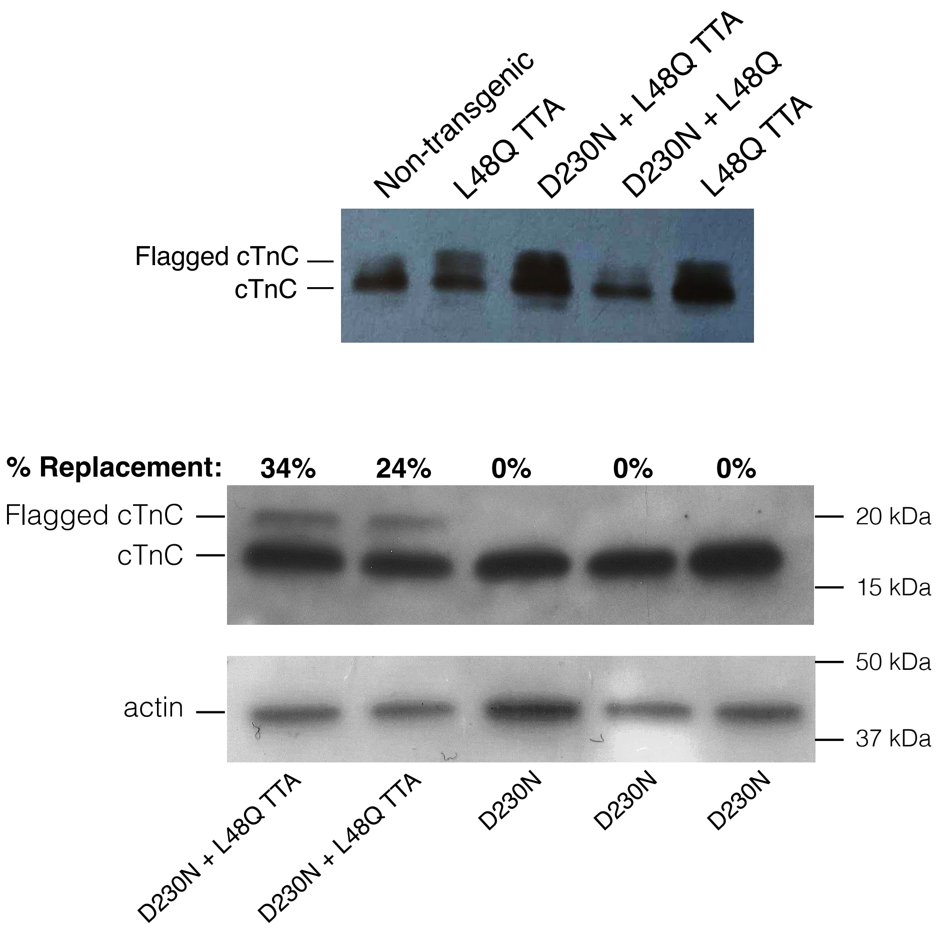

Figure S6. Western blot analyses of L48Q cTnC expression in transgenic mice. *Top Image*: As previously described (Reference 7 in the main text) L48Q cTnC expression is controlled under the tetracycline transactivator construct (TTA). The cTnC antibody shows both the slower migrating flag-epitope-tagged cTnC constructs that were overexpressed and endogenous cTnC. *Bottom Image*: The percent L48Q cTnC replacement was calculated using densitometry analysis of the cTnC bands compared to the flagged cTnC bands. On average, DTG hearts (D230N + L48Q TTA) have ~30% L48Q cTnC and D230N Tm hearts have 0%. Actin was used as a loading control.

Supplemental Tables

Table S1. Fold-changes in parameters used in the Negroni-Lascano model to simulate D230N twitches with different inotropic targets.

Simulated Twitch	Y_b	Z_b	Y_r	Z_r	f	g	Z_p	Y_p	g_d	Z_q	Y_q
WT	1	1	1	1	1	1	1	1	1	1	1
D230N	1	1	1	1	0.435	1	1	1	1	1	1
D230N + XB modulator	1	1	1	1	0.435	0.9–0.5	0.9–0.5	2–10	1	1	1
D230N + Ca^{2+} modulator	2–9	0.9–0.5	0.9–0.5	2–10	0.435	1	1	1	1	1	1

See Figure S2 for schematic of the model. XB: cross-bridge.

Table S2. Mechanics measurements of cardiac muscle preparations corresponding to Figure 4 in the main text.

Intact Trabeculae	WT (n = 6)	L48Q (n = 5)	D230N (n = 7)	DTG (n = 8)
T_{peak} (kPa)	61.9 ± 4.6	55.5 ± 3.0	28.3 ± 2.1$^{*,+}$	54.1 ± 4.9
TT_P (ms)	100.3 ± 5.1	94.1 ± 3.2	91.7 ± 5.2	85.7 ± 2.3
RT_{50} (ms)	62.4 ± 3.4	79.6 ± 11.5^{+}	51.3 ± 3.0	54.6 ± 2.5
RT_{90} (ms)	108.3 ± 5.7	136.7 ± 12.8^{+}	88.9 ± 6.6	93.4 ± 4.4
TI (T·ms)	0	3.1x10^3	–7.0x10^3	–2.7x10^3
**Permeabilized Muscle Strips**	**(n = 13)**	**(n = 10)**	**(n = 16)**	**(n = 9)**
T_{max} (kPa)	37.8 ± 5.0	28.8 ± 6.5	49.4 ± 6.6	36.0 ± 3.8
pCa_{50}	5.57 ± 0.02	5.79 ± 0.05$^{*,+}$	5.43 ± 0.04$^{*,+}$	5.58 ± 0.02
n_H	2.80 ± 0.18	2.95 ± 0.42	3.12 ± 0.10	3.63 ± 0.51

T_{peak}: Peak Twitch Tension; TT_P Time to T_{peak} relative to the electrical stimulus; RT_{50}: time to 50% relaxation (relative to T_{peak}); RT_{90}: time to 90% relaxation (relative to T_{peak}); pCa_{50}: pCa at half-maximum steady-state tension; n_H; Hill coefficient as a result of the fit. $^*p < 0.05$ compared to WT and $^+p < 0.05$ compared to DTG using a one-way ANOVA with a Tukey post-hoc test of significance. Values represent mean ± SEM, with the exception of the TI, which was calculated based on the average twitch trace for each genotype.

Table S3. Echocardiographic measurements corresponding to Figure 5 in the main text.

**WT**	**2 months (n = 9)**	**3 months (n = 9)**	**4 months (n = 7)**	**5 months (n = 4)**
FS (%)	39.1 ± 0.9	39.1 ± 0.7	38.0 ± 1.3	38.2 ± 1.2
EF (%)	70.3 ± 1.1	70.3 ± 0.9	68.8 ± 1.7	69.3 ± 1.5
$LVID_D$ (mm)	3.74 ± 0.10	3.85 ± 0.10	3.62 ± 0.05	3.69 ± 0.10
$LVID_S$ (mm)	2.28 ± 0.08	2.34 ± 0.06	2.25 ± 0.07	2.28 ± 0.08
**L48Q**	**2 months (n = 4)**	**3 months (n = 5)**	**4 months (n = 4)**	**5 months (n = 4)**
FS (%)	37.4 ± 1.1	39.8 ± 0.7	42.2 ± 2.6^{+}	42.6 ± 0.9$^{+,\#}$
EF (%)	68.6 ± 1.4	71.8 ± 0.9	74.0 ± 2.9^{+}	74.6 ± 1.1$^{+,\#}$
$LVID_D$ (mm)	3.30 ± 0.06*	3.21 ± 0.10$^{*,+}$	3.39 ± 0.03^{+}	3.53 ± 0.12$^{\#}$
$LVID_S$ (mm)	2.07 ± 0.06	1.93 ± 0.08$^{*,+}$	1.96 ± 0.10^{+}	2.03 ± 0.09$^{+,\#}$
**D230N**	**2 months (n = 7)**	**3 months (n = 8)**	**4 months (n = 6)**	**5 months (n = 7)**
FS (%)	34.5 ± 1.0*	32.9 ± 1.3$^{*,+}$	27.2 ± 2.5$^{*,+}$	29.1 ± 1.1$^{*,+,\#}$
EF (%)	64.1 ± 1.4*	61.6 ± 2.0$^{*,+}$	53.0 ± 4.1$^{*,+}$	56.3 ± 1.6$^{*,+,\#}$
$LVID_D$ (mm)	3.99 ± 0.09^{+}	4.11 ± 0.14^{+}	4.11 ± 0.18*	4.32 ± 0.08$^{*,+,\#}$
$LVID_S$ (mm)	2.61 ± 0.08$^{*,+}$	2.77 ± 0.13$^{*,+}$	3.02 ± 0.23$^{*,+}$	3.06 ± 0.07$^{*,+,\#}$
**DTG**	**2 months (n = 13)**	**3 months (n = 12)**	**4 months (n = 14)**	**5 months (n = 8)**
FS (%)	36.9 ± 0.8	37.2 ± 0.8	33.5 ± 0.8	34.2 ± 1.5
EF (%)	67.5 ± 1.1	67.8 ± 1.1	62.8 ± 1.2	63.7 ± 2.0
$LVID_D$ (mm)	3.65 ± 0.06	3.72 ± 0.06	3.86 ± 0.07	3.75 ± 0.11
$LVID_S$ (mm)	2.31 ± 0.05	2.34 ± 0.06	2.57 ± 0.06	2.46 ± 0.06

FS: Fractional Shortening; EF: Ejection Fraction; $LVID_D$: Diastolic left-ventricular inner diameter; $LVID_S$: Systolic left-ventricular inner diameter. $^{*}p < 0.05$ compared to WT and $^{+}p < 0.05$ compared to DTG using a one-way ANOVA with a Tukey post-hoc test of significance. $^{\#}p < 0.05$ comparing ages 2 to 5 months within genotype groups using an unpaired student's t-test. Values represent mean ± SEM.
